# A viscosity-tunable microgel-in-sol phase for localized inner ear delivery of small hydrophilic drug

**DOI:** 10.1016/j.mtbio.2026.103590

**Published:** 2026-08-24

**Authors:** Sumin Kim, Jee Hyun Kang, Seong Su Won, Keum-Jin Yang, Subin Kim, Dong-Kee Kim, Mikyung Shin

**Affiliations:** a Department of Intelligent Precision Healthcare Convergence, Sungkyunkwan University, Suwon, 16419, Republic of Korea; b Center for Neuroscience Imaging Research, Institute for Basic Science (IBS), Suwon, 16419, Republic of Korea; c Department of Otolaryngology, College of Medicine, The Catholic University of Korea, Daejeon, 34943, Republic of Korea; d Clinical Research Institute, Daejeon St. Mary's Hospital, Daejeon, Republic of Korea; e Department of Otolaryngology, College of Medicine, Soonchunhyang University College of Medicine, Cheonan, 31151, Republic of Korea; f Department of Biomedical Engineering, Sungkyunkwan University, Suwon, 16419, Republic of Korea

**Keywords:** Sodium thiosulfate, Cisplatin-induced hearing loss, Intratympanic injection, Microgels, Viscosity-tunable, Localized delivery

## Abstract

Cisplatin-induced ototoxicity leads to irreversible hearing loss in approximately 50% of treated patients, largely due to the cochlea's exceptionally poor cisplatin clearance compared with other organs. Although sodium thiosulfate (STS), a hydrophilic small molecule, is an established otoprotectant, achieving therapeutically sufficient cochlear concentrations remains challenging because systemic administration is limited by the blood–labyrinth barrier. Here, we report a viscosity-tunable microgel-in-sol system based on hyaluronic acid (HA) that enables sustained, localized delivery of STS to the inner ear via intratympanic injection. The STS-loaded microgel was readily injectable through 31 G needles and provided a six-fold extension in drug release duration compared with free STS solution. However, direct microgel injection caused significant occlusion of the middle ear cavity due to swelling and poor clearance. To overcome this limitation, we developed a microgel-in-sol formulation composed of an HA solution and microgel at a 3:1 volumetric ratio. This formulation exhibited concentration-dependent, lubricant-like viscosity while maintaining effective drug delivery and preserving auditory function, as confirmed by in vivo auditory brainstem response (ABR) measurements. Overall, this microgel-in-sol strategy represents a biocompatible, minimally invasive approach for localized inner-ear drug delivery.

## Graphical abstract

**Figure:**
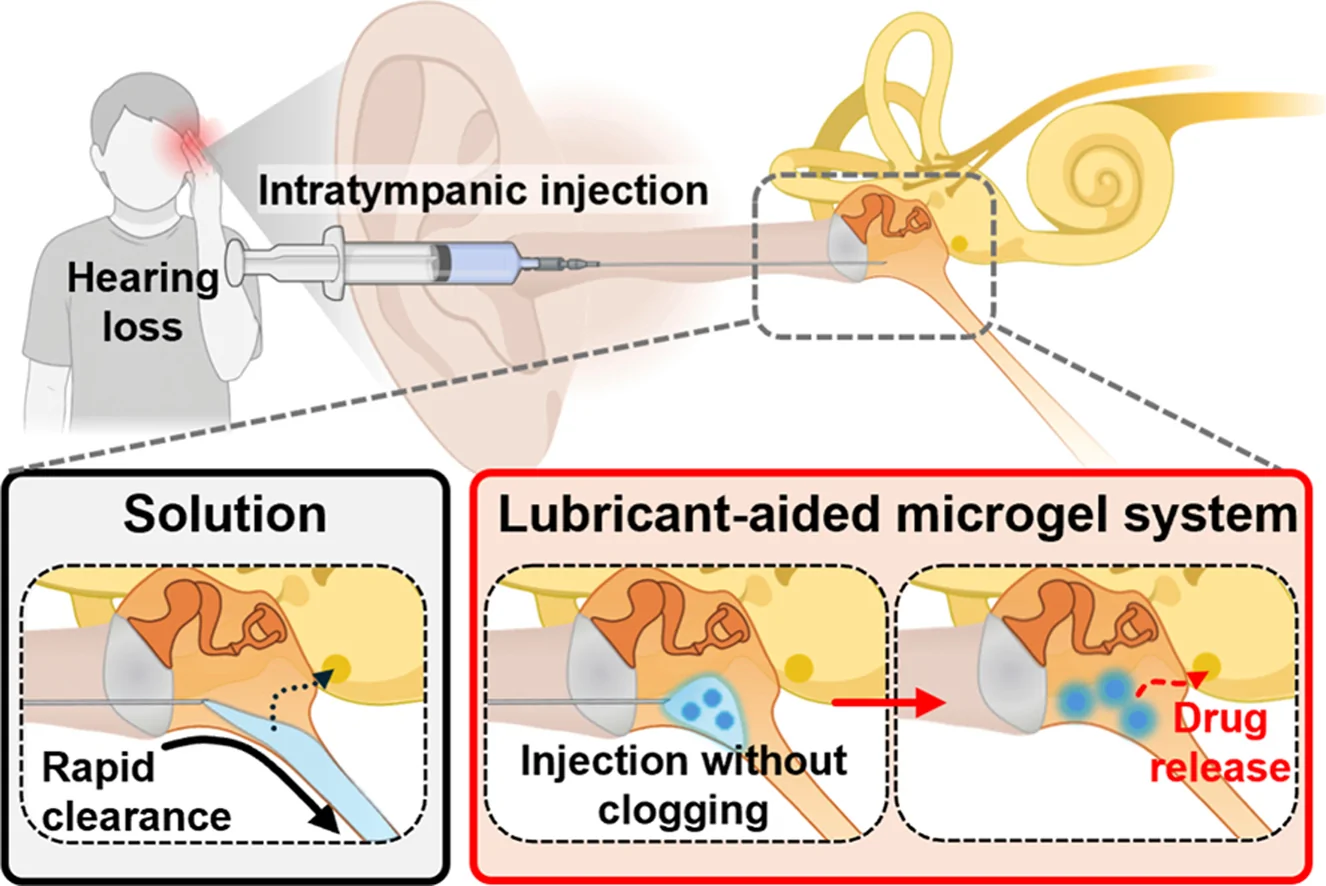

## 1 Introduction

Cisplatin is widely used as a chemotherapeutic agent for the treatment of a broad range of cancers, including leukemia, lymphoma, breast, testicular, ovarian, cervical, and head and neck cancers, as well as sarcomas [[1], [2], [3]]. However, a critical drawback is that cisplatin accumulates in the inner ear and induces ototoxicity [4,5]. Unlike most organs, where cisplatin is detected and eliminated within days to weeks following administration, the cochlea retains cisplatin for months to years after treatment, making it far more vulnerable to cumulative toxic exposure and resulting in irreversible hearing loss [6,7]. Among the protective agents to prevent cisplatin-induced ototoxicity, corticosteroids (e.g., dexamethasone) and thiol-based antioxidants/detoxifying agents (e.g., sodium thiosulfate (STS)) are considered as two major classes [8,9]. To date, STS has been most extensively developed clinically, owing to its ability to directly inactivate cisplatin through sulfhydryl-mediated covalent binding while suppressing reactive oxygen species (ROS) accumulation in outer hair cells [[10], [11], [12]]. Nevertheless, its effective delivery to the cochlea still remains limited by the blood-labyrinth barrier (BLB) [[13], [14], [15], [16]]. Consequently, local intratympanic (IT) administration is required for enhancing cochlear drug exposure [17,18].

Although early clinical studies have focused on the safety and feasibility of IT delivery of STS alone, free STS exhibits a perilymph half-life of only 0.74 h [19]. Various local drug delivery platforms including phospholipid [20] and polymeric nanocarriers [[21], [22], [23]] have been recently developed to improve inner ear drug delivery through their surficial engineering with different electrostatic charge or functional moieties, yet the highly hydrophilic nature of STS makes its efficient encapsulation within the hydrophobic core of the nanoparticles challenging. In this regard, hydrogel matrices with high water contents can be considered as intratympanic delivery platforms [24,25] because they prolong residence within the middle ear, thereby sustaining drug release at the round window membrane and enhancing cochlear drug exposure [26]. However, the conventional bulk hydrogel form is poorly suited for IT injection, which requires passing through the tympanic membrane. To minimize invasiveness to the membrane, an extremely fine needle must be used. Indeed, studies employing bulk hydrogel-based IT delivery in guinea pigs have generally required relatively large-bore needles (∼22 G) to prevent clogging of highly viscous formulations within the needle [27,28]. In contrast, small-sized microgels enable smooth injection through a much finer 31 G needle while maintaining the advantages of a hydrogel-based delivery system. Because a 31 G needle has an outer diameter of approximately 0.25 mm, compared with ∼0.71 mm for a 22 G needle, its use may reduce mechanical trauma to the tympanic membrane during IT administration [29,30]. Also, in clinical practice, IT injection for sudden sensorineural hearing loss — as recommended by the American Academy of Otolaryngology–Head and Neck Surgery (AAO-HNS) Clinical Practice Guideline — typically employs 400 - 800 μL per injection, up to 4 injections over a 2-week period, to fill the human middle ear space [[31], [32], [33]]. Although the total volume of the mouse middle ear cavity has not been formally reported, injection volumes in murine models are as low as 2 μL [34], meaning the importance of volumetric control in this confined space.

**Fig. 1:**
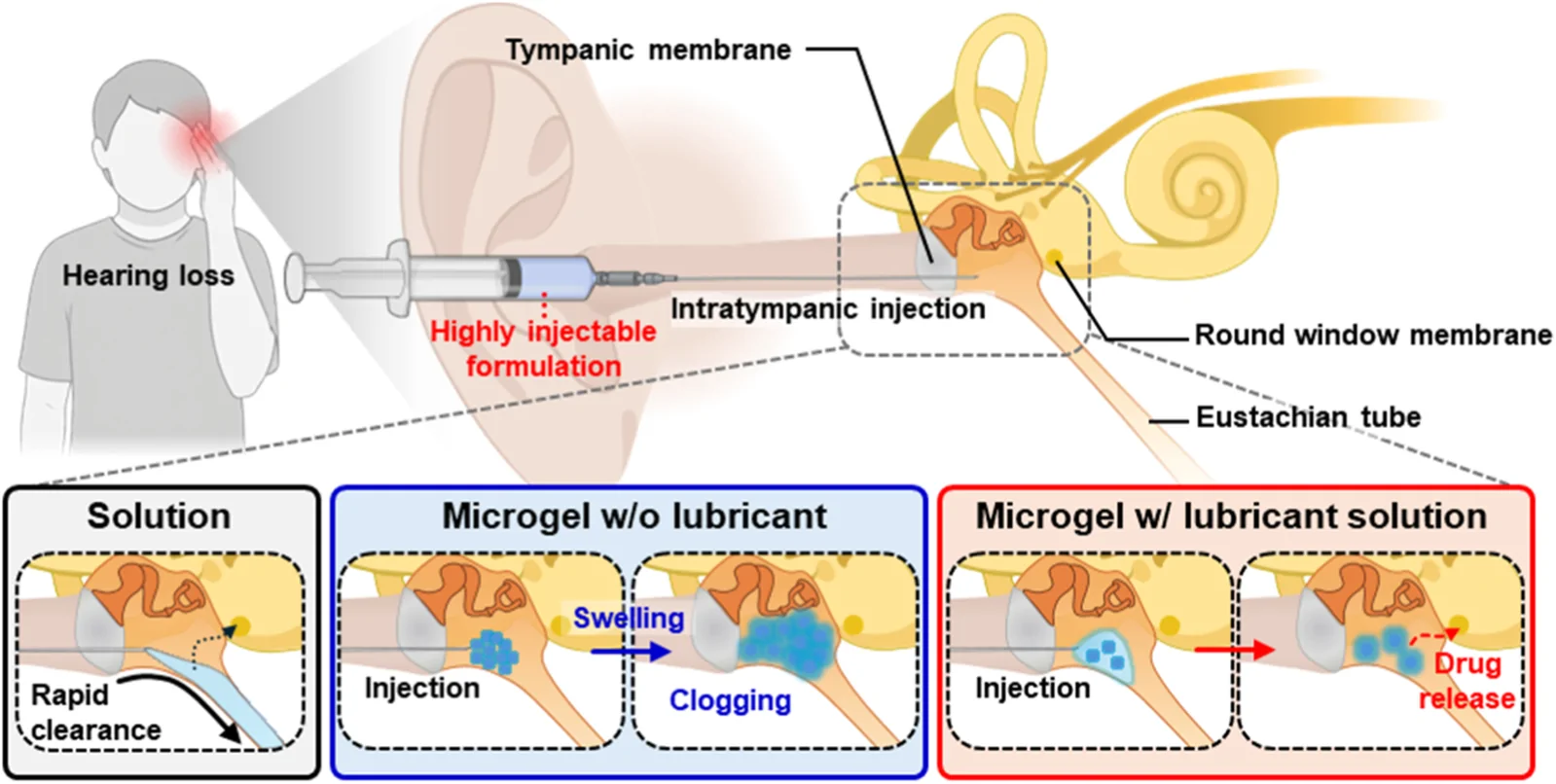
Overall schematic description for injection of the microgel-in-sol phase with drug loading and lubricant carrier (red) for inner ear drug delivery compared to that of drug solution alone (black) and the drug-loaded microgels (blue). (For interpretation of the references to color in this figure legend, the reader is referred to the Web version of this article.)

Herein, we report a viscosity-tunable microgel-in-sol phase that is designed to overcome the limitations of existing IT delivery platforms by combining prolonged middle ear retention and preservation of middle ear function without temporary cavity occlusion. (Fig. 1, red square). When microgels alone were applied to the middle ear cavity, it was found that swelling of the microgel physically occludes the ear in a manner analogous to an earplug (Fig. 1, blue square), thereby affecting hearing response [35]. Thus, a microgel-in-sol phase was prepared mixing the microgel with a viscous HA solution as a lubricant carrier, which was highly viscosity-tunable enough to inject extremely small volume of the materials into the ear cavity. When compared to rapid clearance of STS alone after IT injection (Fig. 1, black square), the gel-in-sol phase with STS loading retained high drug concentration in the ear at 1 h. This approach prevents rapid dissociation of the microgel while avoiding physical occlusion of the middle ear cavity and preserving auditory function and enables the desired therapeutic effect of STS.

## 2 Results and discussion

### 2.1 Fabrication and characterization of HA microgels

**Fig. 2:**
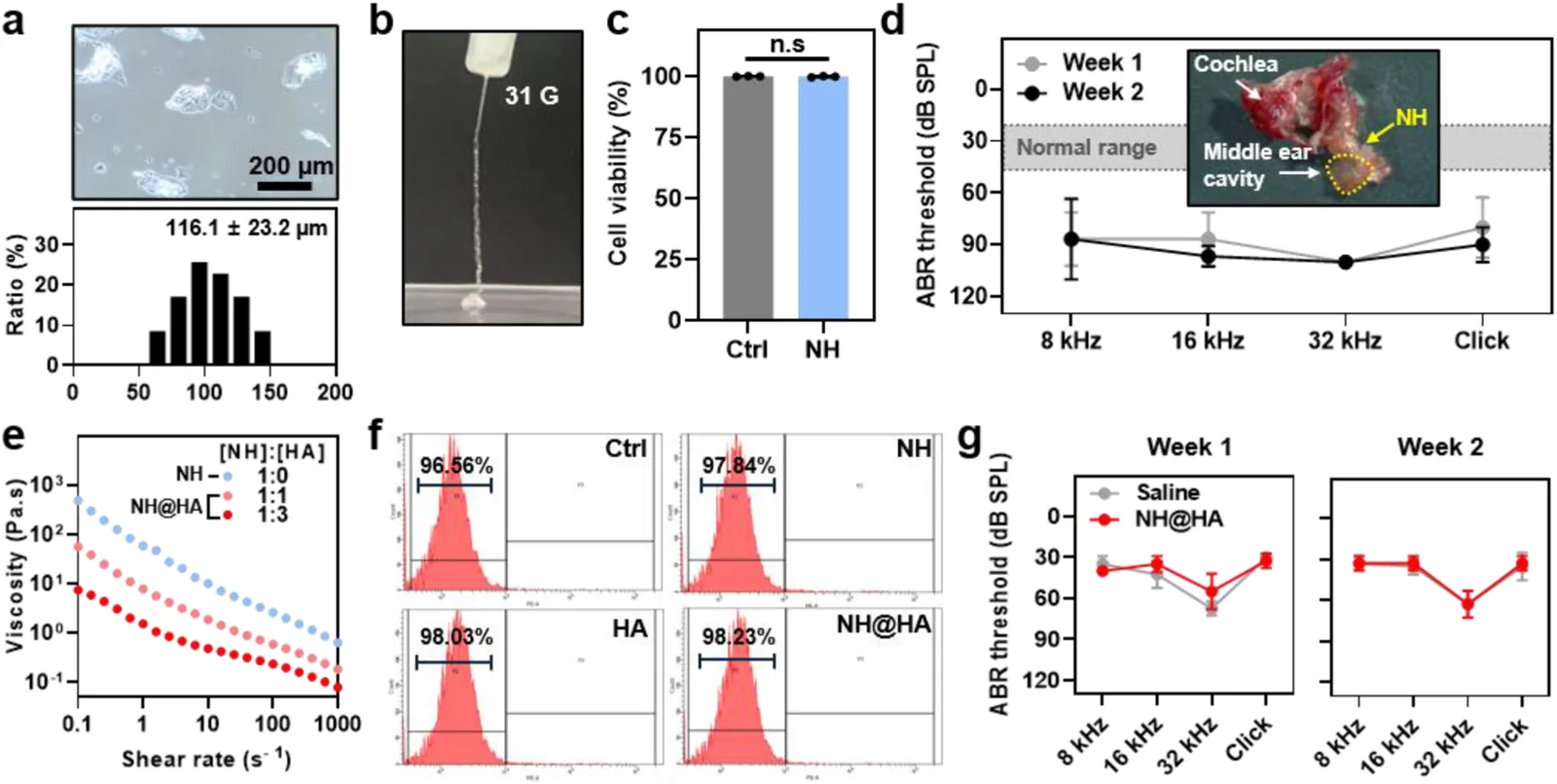
**Characterization of HA microgels (NH) and the microgel-in-sol phase, NH@HA, as an inner ear drug delivery platform.** (a) Optical microscopy image of NH (top) and corresponding size distribution histogram (bottom). (b) Photograph demonstrating successful injection of NH through a 31 G needle. (c) Cell viability of hair cell (HEI-OC1) cultured with NH compared to the control group. Unpaired two-tailed *t*-test. n.s, ‘not significant’. (d) ABR measurements at Week 1 and Week 2 after injection of NH alone, and a representative photograph showing physical clogging of the middle ear cavity caused by swelling of the injected NH. The grey shaded area indicates the normal ABR threshold range. (e) Shear viscosity profiles of NH (1:0), NH@HA, 1:1 and 1:3 ([NH]:[HA]), at shear rates from 0.1 to 1000 s^−1^, demonstrating a decrease in apparent viscosity with increasing HA content. (f) Flow cytometry analysis of hair cell viability following treatment with NH, HA, and NH@HA. Ctrl for non-treatment group. (g) ABR measurements at Week 1 and Week 2 after injection of the NH@HA (red) or saline (grey), confirming no significant impairment of auditory function. (For interpretation of the references to color in this figure legend, the reader is referred to the Web version of this article.)

As mentioned above, the inner ear is a highly inaccessible anatomical site, a minimally invasive drug delivery system is necessarily required. In this regard, a microgel-based system is beneficial for achieving minimally invasive and localized drug delivery to inner ear. As reported previously, jammed microgels – which is densely packed microgels under vacuum filtration – are highly injectable, extrudable, and printable [[36], [37], [38]]. First, we fabricated HA microgels using norbornene-conjugated hyaluronic acid (NorHA) which is photo-crosslinkable via thiol-ene reactions, enabling facile tuning of mechanical properties of the hydrogel and granulation through a mesh-fragmentation process [39]. NorHA was synthesized with a degree of norbornene substitution of approximately 30% and NorHA microgels (NH) was fabricated with a size of approximately 116 ± 23.2 μm passing through an iron mesh (Fig. 2a, Fig. S1). Their jammed hydrogels were injectable using a 31G needle with the inner diameter of approximately 120 μm (Fig. 2b), indicating optimal formulations for localized inner ear drug delivery. Even though the NH microgel diameter was comparable to the needle inner diameter, a certain degree of physical rearrangement of the microgel might contribute to smooth injection through a fine needle [36].

Thanks to high biocompatibility of HA, the NH did not show any cytotoxicity in both fibroblasts (e.g., L929) and auditory hair cells (e.g., HEI-OC1) (Fig. 2c, Fig. S2). Such great cytocompatibility is considerably important in inner ear applications where cochlear hair cells possess very limited regenerative capacity once damaged [40,41]. Continuing in vitro test, we injected the jammed microgels into the middle ear cavity in vivo for investigating auditory function. While normal ABR thresholds for 8-week-old mice typically remain around 30 dB SPL across frequencies [42,43], the NH-injected group exhibited markedly elevated thresholds of approximately 90 dB that persisted for over 2 weeks (Fig. 2d). Rather than reflecting auditory impairment, this increase was attributed to the swollen microgels physically obstructing sound transmission within the middle ear cavity. Under high moisture of the middle ear cavity with non-favorable clearance, the microgel was swollen and acted as a physical barrier against hearing assessments, like an earplug even though histological images stained by hematoxylin and eosin (H&E) showed negligible immune response (Fig. 2d, Fig. S3). Thus, we hypothesized that a viscous solution as a lubricant, not a gel phase, is needed for normal auditory function and well-clearance within the ear.

### 2.2 Preparation of the HA microgel-in-sol phase

To address this limitation, we introduced a high-molecular-weight HA solution (e.g., 700 kDa) as a lubricant phase and dispersed NH into the HA solution (named NH@HA) by varying their volumetric ratio, 1:0, 1:1, and 1:3 ([NH]:[HA]) (Fig. 2e, Fig. S4). The viscous HA solution was selected instead of a low-viscosity liquid to stably maintain the microgel-in-sol formulation. Additionally, the microgel-in-sol phase, NH@HA, preserved controllable shear viscosity. Through the rheological analysis of NH or NH@HA, the NH@HA exhibited lower shear viscosity at all shear rates than that of NH alone as the HA proportion increased (Fig. 2e). Similarly, their storage moduli were lower and tan δ values were higher than those of NH alone (Fig. S4). From these results regarding viscosity and injectability, the NH@HA with a ratio of 1:3 was chosen for further in vitro and in vivo studies. The NH@HA also showed no adverse cytotoxicity (viability = 98.23%) in auditory hair cells (e.g., HEI-OC1) (Fig. 2f) and there was no significant difference for hearing assessments in both groups, saline and NH@HA, after IT injection (Fig. 2g). Taken together, this microgel-in-sol phase can be utilized as a viable drug reservoir without occlusion of the middle ear cavity.

### 2.3 Protection effect of STS-loaded NH@HA against cisplatin-induced ototoxicity

STS has been recently highlighted as a hydrophilic ionic compound for protecting cochlear hair cells under systemic administration of cisplatin due to its chelating capability with the antitumor agent, forming biologically inactive platinum-thiosulfate complexes that reduce free platinum exposure to the cochlea [11]. To verify the feasibility of the NH@HA as such hydrophilic STS reservoir for prevention of cisplatin-induced ototoxicity, STS could be loaded into the NH@HA through concentration-driven diffusion into dried NH (Fig. S5a). After lyophilization of NH, the microgels were rehydrated in a STS solution and the drugs were encapsulated into the NH core. There was no significant change in rheological properties of the NH before and after STS loading (Fig. S5b).

**Fig. 3:**
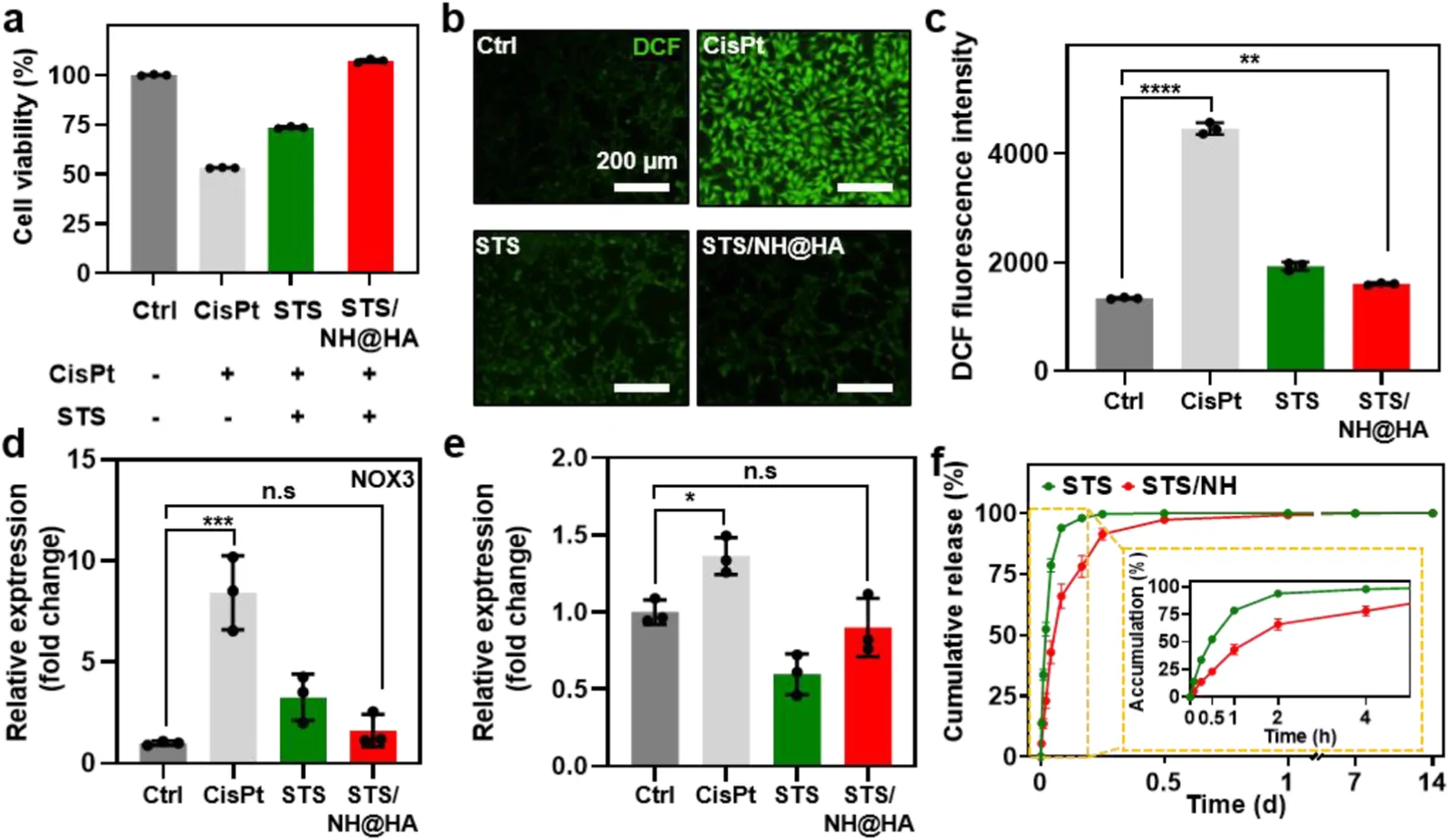
**Protective effects of STS-loaded NH@HA against cisplatin-induced oxidative stress in HEI-OC1 cells.** (a) In vitro viability (%) of HEI-OC1 cells following treatment with cisplatin (CisPt) alone or in combination with either STS solution (STS) or STS-loaded NH@HA (STS/NH@HA). (b) Representative fluorescence images of intracellular ROS detected by DCFH-DA staining in HEI-OC1 cells after treatment of each material. Scale bar, 200 μm. (c) Quantitative analysis of DCF fluorescence intensity in each group. (d) Quantitative real-time PCR (qPCR) analysis of NOX3 mRNA expression. (e) Densitometric quantification of NOX3 protein expression, normalized to β-actin. (f) *In vitro* cumulative release profile of STS from free solution (black line) and encapsulated with NH formulation (red line). The inset shows an enlarged view of the release profiles for initial 5 h. One-way ANOVA, ns for “not significant,” **P < 0.01, ***P < 0.001, and ****P < 0.0001. (For interpretation of the references to color in this figure legend, the reader is referred to the Web version of this article.)

Regarding non-cytotoxic as well as therapeutically effective concentration ranges of STS by itself against cisplatin exposure, HEI-OC1 cells were viable (higher than 70%) at the pre-treated STS concentration ranging from 1 to 20 mM under co-treatment of cisplatin (20 μM) (Fig. S6). Based on these findings, 10 mM of STS was selected to be loaded into the NH for further in vitro experiments, as this concentration provided a favorable balance between cytoprotection and cytocompatibility. Also, STS molecules released from the NH@HA were effective as a protecting agent against cisplatin-induced oxidative stress (Fig. 3a). STS-loaded NH@HA (Fig. 3a–red) showed greater cytoprotective efficacy than STS alone (green). Meanwhile, HA alone could slightly restore cell viability and reduce intracellular ROS levels compared with the cisplatin-treated group (Fig. S7), suggesting that HA may exert modest cytoprotective and antioxidant effects. These findings are consistent with previous reports demonstrating that high-molecular-weight HA attenuates oxidative stress and suppresses apoptosis [44], while also exhibiting anti-inflammatory properties [45]. Although the protective effect of HA alone was lower than that of STS-loaded NH@HA, these findings suggest that the overall cytoprotective effect of the NH@HA system likely reflects contributions from both the intrinsic biological activity of HA and its sustained-release characteristics.

Particularly, cisplatin-induced ototoxicity originates from oxidative stress. That is, increases of intracellular reactive oxygen species (ROS) levels induce apoptosis of the cochlear cells. In this regard, it is important to investigate ROS scavenging effect of the treated drug, STS (or STS-loaded NH@HA). In fluorescence images with 2′,7′-dichlorodihydrofluorescein diacetate (DCFH-DA) staining, cisplatin treatment remarkably increased ROS generation in the cells, yet STS and STS-loaded NH@HA decreased the ROS level (Fig. 3b). In addition to that, ROS quantitative analysis through 2′,7′-dichlorofluorescein (DCF) assay corresponds to the images in all groups (Fig. 3c). To determine whether the reduction in oxidative stress was associated with changes in redox-related signaling, NOX3 expression was analyzed at both the mRNA and protein levels using quantitative real-time PCR and Western blotting (Fig. 3d,e, Fig. S8). Cisplatin treatment significantly upregulated NOX3 expression when compared with normal cells, whereas STS and STS-loaded NH@HA treatment attenuated cisplatin-induced NOX3 upregulation. Given that NOX3 is the predominant NADPH oxidase isoform in the cochlea and a key mediator of ROS generation [46], its downregulation supports that STS-loaded NH@HA attenuates oxidative stress at the molecular level rather than merely reducing intracellularly accumulated ROS. All these findings clearly suggest that STS released from NH@HA provides ROS scavenging effect against cisplatin-induced oxidative stress.

Sustained STS release characteristics from the NH system was investigated prior to in vivo application (Fig. 3f). The high concentration of free HA in the NH@HA formulation exhibits an absorption at the wavelength ranging from 200 to 250 nm (Fig. S9), which can interfere with the absorption of STS during the initial release period. Thus, we evaluated STS release from NH alone. The STS solution alone exhibited immediate drug diffusion within ∼4 h (Fig. 3f, black), in contrast, STS was released from the NH formulation over 24 h (red). This prolonged release of STS from the microgel is noteworthy when considering that STS IT injection shows rapid clearance kinetics in which cochlear is exposed to the drug only for a few hours [26,47]. The microgel-mediated STS delivery might therefore help overcome a key limitation of existing local STS injection approaches and expand the therapeutic time for otoprotection. In in vivo experimental settings, cochlear thiosulfate concentrations were determined using high-performance liquid chromatography (HPLC)-inductively coupled plasma-mass spectrometry (ICP-MS/MS) after IT administration of STS alone or STS-loaded NH@HA (Fig. S10). Although STS levels decreased over time in both groups, NH@HA showed higher mean STS concentrations during the measured 1–3 h period, representing the early phase following IT administration, compared with free STS. These findings indicate that NH@HA enhances cochlear STS exposure during the early period following IT administration. Notably, while the in vitro STS release profile from NH indicated sustained drug release, cochlear STS concentrations were already comparable between the STS and STS/NH@HA groups at 6 h. This finding may be attributable to the rapid clearance of STS released from the hydrogel system through several in vivo processes, including middle-ear clearance, transport across the round window membrane, perilymph turnover, and subsequent vascular elimination.

Collectively, the NH@HA enhances local retention and continuous exposure of STS to cochlear tissues, thereby suppressing NOX3-associated oxidative stress and ultimately protecting auditory cells against cisplatin-induced ototoxicity.

### 2.4 In vivo therapeutic effect of STS-loaded NH@HA against cisplatin-induced ototoxicity

**Fig. 4:**
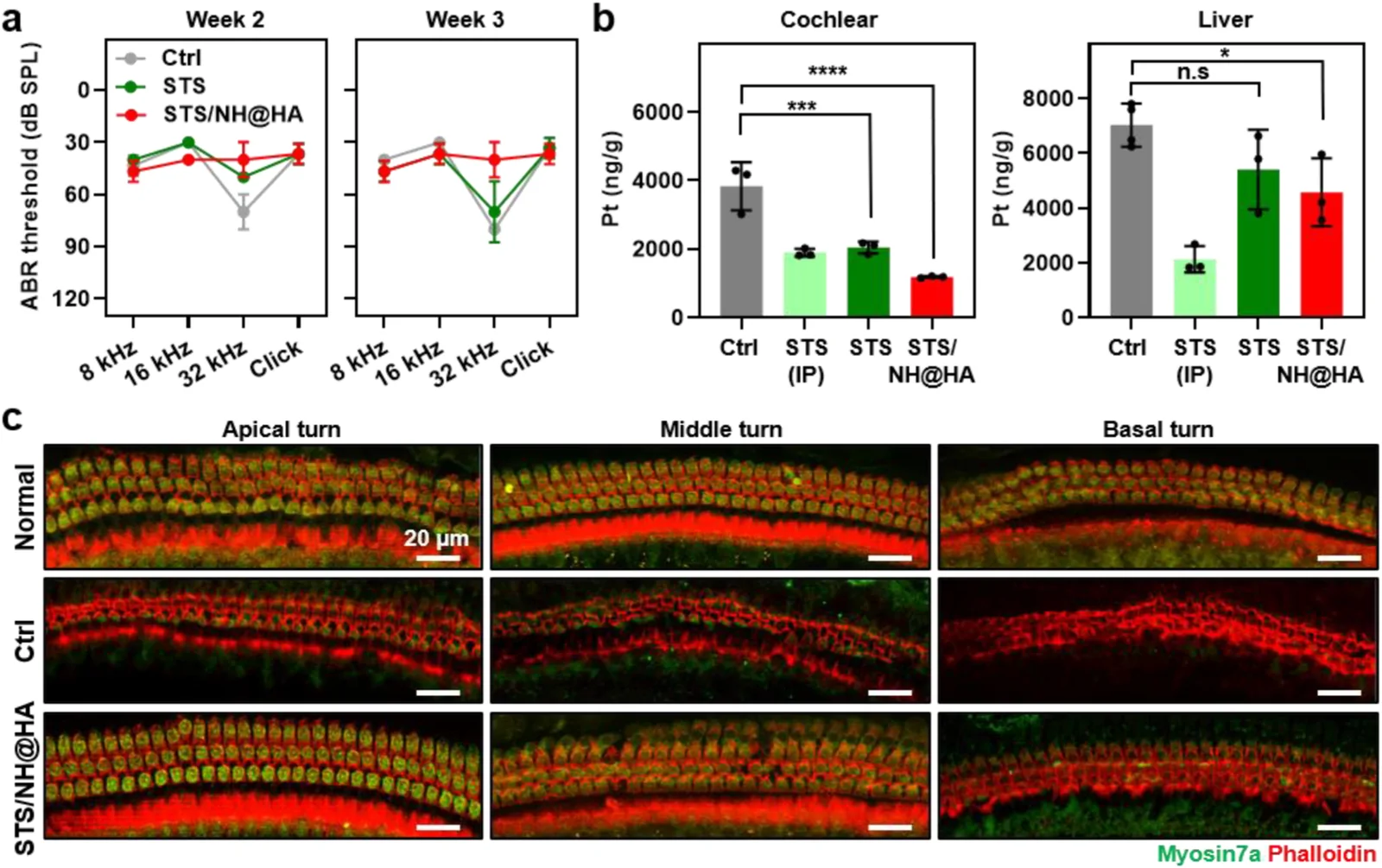
**In vivo otoprotective efficacy of STS-loaded NH@HA.** (a) ABR thresholds measured at Week 2 and 3 after systemic administration of cisplatin. ‘Ctrl’ for non-treatment, ‘STS’ for IT injection of STS solution (500 mM), and ‘STS/NH@HA’ for IT injection of STS (500 mM)-loaded NH@HA prior to cisplatin administration (16 mg/kg, i.p.). ABR thresholds assessed at 8, 16, and 32 kHz and click stimuli. (b) Platinum concentrations in the cochlea and liver following systemic or IT administration of STS or STS/NH@HA. (c) Representative whole-mount immunofluorescence images of the organ of Corti from the apical, middle, and basal turns. Hair cells were labeled with Myosin7a (green), and F-actin was visualized with phalloidin (red). Cisplatin treatment disrupted hair cell architecture, particularly in the basal turn, whereas NH@HA-mediated STS delivery preserved cochlear morphology. Scale bars, 20 μm. One-way ANOVA, n.s for “not significant,” *P < 0.05, ***P < 0.001, and ****P < 0.0001. (For interpretation of the references to color in this figure legend, the reader is referred to the Web version of this article.)

Finally, we evaluated in vivo therapeutic efficacy of STS-loaded NH@HA for protecting cochlear hair cells against cisplatin (Fig. 4). The STS IT administration with a concentration of 100, 500 and 1000 mM represented ABR thresholds in the range from 25 to 50 dB over 3 weeks similar to normal level, which indicated no cochlear toxicity (Fig. S11). Although relatively high administration concentrations of STS are required for in vivo intratympanic delivery, only a fraction of the administered drug reaches the cochlea because most of molecules are rapidly diffused across the middle-ear cavity and round window membrane. That is, its administered dose does not directly match to the drug concentration within cochlear tissues. Comparable cochlear exposure would be difficult to achieve through systemic administration. Considering the biological safety of STS IT delivery system for otoprotection, 500 mM STS was selected for the subsequent in vivo experiments [13]. In ABR analysis, STS alone and STS-loaded NH@HA exhibited in vivo otoprotective effects under systemic administration of cisplatin (Fig. 4a). At 2 weeks after cisplatin administration, the control group (e.g., non-treatment) exhibited elevated ABR thresholds, particularly at high frequencies (32 kHz), whereas STS or STS-loaded NH@HA groups showed partial preservation of hearing function. The critical effect was observed at 3 weeks in which the only NH@HA group showed pronounced protection and preservation of auditory function, especially at 32 kHz. Additionally, NH@HA without STS encapsulation did not alter cisplatin-induced ABR threshold shifts at either 2 or 3 weeks (Fig. S12), indicating a negligible effect of the carrier itself on in vivo functional hearing protection other than that of in vitro results (Fig. S7).

For further discussion, the great protection effect of STS-loaded NH@HA at 3 weeks does not imply prolonged STS release or cochlear retention over several weeks. Rather, sustained release from NH@HA can maintain local STS availability during the early period after administration, thereby reducing initial cochlear injury and limiting its subsequent progression. Given the prolonged retention of cisplatin-derived platinum in the cochlea and the progressive nature of cisplatin-induced ototoxicity [6,48], improved early STS availability might result in a protective effect that becomes more apparent at later time points.

Furthermore, considering that severe cisplatin accumulation into the tissue affects toxicity, we investigated platinum distribution in cochlear and liver tissues through ICP-MS anlaysis(Fig. 4b). In the cochlea, platinum accumulation was reduced in all STS-treated animals compared with controls. Particularly, cochlear platinum levels were lower in the NH@HA group than STS solution, which may be related to the prolonged cochlear retention of STS achieved by the microgel formulation. Because cisplatin is usually applied for its antitumor activity through systemic route, we also examined hepatic platinum accumulation to determine whether local STS administration affects systemic cisplatin biodistribution. In the liver, systemic STS administration markedly reduced platinum accumulation compared with controls, indicating substantial systemic interaction with cisplatin. In contrast, STS IT delivery largely preserved hepatic platinum levels. Although NH@HA-mediated IT delivery produced a moderate reduction in liver platinum levels, hepatic platinum accumulation remained substantially higher than that observed following systemic STS administration. These findings suggest that local STS delivery effectively reduces cochlear platinum accumulation yet does not affect biodistribution of cisplatin by itself, potentially preserving its antitumor efficacy, after systemic administration.

To further assess histological preservation of the cochlea, immunofluorescence staining was performed using Myosin7a and phalloidin to visualize hair cells and actin cytoskeletal organization (Fig. 4c). Cisplatin-treated mouse exhibited disrupted hair cell architecture, particularly in the basal turn, whereas STS-loaded NH@HA hydrogels preserved hair cell morphology across the apical, middle, and basal turns. The number of outer hair cells (OHCs) were also quantitatively analyzed over a standardized cochlear length of 100 μm in each cochlear turn (Fig. S13). Consistent with the representative images, cisplatin treatment significantly reduced OHC numbers in the apical, middle, and basal turns, whereas STS-loaded NH@HA significantly preserved OHC survival in all cochlear regions. Notably, OHC counts in the STS-loaded NH@HA group were not significantly different from those in the normal group. Outer hair cell organization and supporting structures remained largely intact following NH@HA-mediated IT delivery, consistent with the functional protection observed in ABR measurements. The concordance between preserved cochlear morphology and improved auditory function indicates that NH@HA-mediated STS delivery provides both structural and functional protection against cisplatin-induced cochlear injury.

Taken together, microgel-mediated STS delivery improves preservation of high-frequency hearing, consistent with enhanced otoprotective efficacy through sustained local drug exposure. The superior preservation of high-frequency hearing might originate from prolonged local availability of STS at the round window membrane, thereby maintaining continuous detoxification of cisplatin and antioxidant activity during the early phase of cochlear injury. Also, STS delivery by the microgel-in-sol phase reduces cochlear platinum accumulation, preserves cochlear architecture, and improves auditory function while minimizing systemic alterations in cisplatin distribution. That is, NH@HA-mediated IT delivery can be a promising strategy for selectively protecting the inner ear without substantially affecting systemic cisplatin disposition.

## 3 Conclusion

In conclusion, we developed a microgel-in-sol phase for delivering small, hydrophilic, and ionic compounds, STS. Particularly, the use of HA solution for the microgel dispersion is helpful for minimally invasive, localized IT injections without physical occlusion of middle ear cavity followed by decreases in auditory assessment. Through in vitro and in vivo evaluations, STS-loaded NH@HA showed excellent biological safety, attenuated cisplatin-induced cytotoxicity, reduced cochlear platinum accumulation, and preserved hair cell architecture and high-frequency hearing without compromising systemic cisplatin efficacy. These findings establish the microgel-in-sol phase as a drug-reservoir platform for otoprotection during cisplatin-based chemotherapy. Further validation in larger animal models and optimization of drug release kinetics for small hydrophilic molecules would be important toward clinical translation.

## 4 Methods

### 4.1 Materials

All reagents were purchased from Sigma-Aldrich otherwise mentioned.

### 4.2 Norbornene conjugated hyaluronic acid (NorHA), NH fabrication and STS encapsulation into the microgel

NorHA was synthesized following a previously reported protocol [39]. Briefly, sodium hyaluronan (60 kDa, Lifecore) was converted to its tetrabutylammonium salt (HA-TBA), then reacted with 5-norbornene-2-carboxylic acid and Boc_2_O in the presence of DMAP in DMSO at 45°C for 18 h. The product was dialyzed against deionized water (DW) with NaCl for 7 days and lyophilized. To fabricate NH, NorHA (4% w/v) was dissolved in DW containing DTT (0.04%) and Irgacure 2959 (0.05%), then crosslinked under UV irradiation (10 mW cm^−2^, 100 s) to form a bulk hydrogel. The bulk hydrogel was extruded through an iron mesh (700 mesh, 700 openings per linear inch) under applied pressure to granulate into microgels. The granulated microgels were re-suspended in DW containing DTT (0.1%) and Irgacure 2959 (0.05%), followed by UV irradiation (10 mW cm^−2^, 3 min) with vigorous pipetting to minimize inter-particle crosslinking. Microgels were then collected by vacuum-driven filtration (0.45 μm PVDF membrane) and lyophilized until use. The size distribution of NH was determined by optical microscopy. Images were acquired and analyzed using ImageJ software to measure the diameter of individual microgels.

For loading of STS, the molecule was dissolved in saline at the desired concentration. Lyophilized NH (25 mg) was then swollen in 1 mL of the STS solution to yield a 2.5% (w/v) microgel suspension overnight, allowing full diffusion of STS throughout the hydrogel network during swelling. No phase separation or residual STS solution was observed after the swelling/diffusion process, indicating homogeneous distribution of the aqueous STS solution within the swollen microgels.

### 4.3 In vitro cell viability test

To assess the cytocompatibility of NH, L929 fibroblasts were seeded in a 24-well plate (5 × 10^4^ cells/well) and co-cultured with NH (50 μL/well) in transwell inserts for 72 h in DMEM supplemented with 10% FBS and 1% penicillin–streptomycin. Cell viability was evaluated using a Live/Dead assay (calcein AM/ethidium homodimer-1; Invitrogen), imaged on a Leica DMi8 fluorescence microscope, and quantified with ImageJ. For HEI-OC1 auditory cells, they were maintained in high-glucose Dulbecco's modified Eagle's medium (DMEM; Gibco, USA) supplemented with 10% fetal bovine serum (FBS; Gibco) and 50 U/mL interferon-γ (IFN-γ). Cells were cultured under permissive conditions at 33°C in a humidified incubator with 10% CO_2_. To evaluate the cytotoxicity of STS, NH formulations, cell viability was assessed using both a colorimetric assay (EZ-CytoX; Daeil Lab Service, Korea) and flow cytometric analysis with propidium iodide (PI) staining. HEI-OC1 cells were seeded into 24-well plates at a density of 3.0 × 10^4^ cells per well and allowed to attach overnight. NH, HA and STS-loaded NH@HA formulations were placed in transwell inserts (0.8 μm pore size; Corning, USA) and positioned above the cell monolayer to allow indirect exposure via diffusion. Following treatment, EZ-CytoX reagent was added to each well according to the manufacturer's instructions and incubated for 2 h at 33°C. Absorbance was measured at 450 nm using an Infinite® 200 PRO multimode microplate reader (Tecan Group Ltd., Männedorf, Switzerland). For flow cytometric analysis, cells were harvested by trypsinization, washed twice with phosphate-buffered saline (PBS), and resuspended in PBS containing 1 μg/mL propidium iodide (PI). Cells were incubated for 15 min at room temperature in the dark. PI-positive cells were quantified using a FACSCanto™ II flow cytometer (BD Biosciences, San Jose, CA, USA). The percentage of PI-negative cells was quantified.

### 4.4 Rheological analysis

The viscosity of NH and NH@HA were measured using a Discovery Hybrid Rheometer 2 (TA Instruments, USA) with a 20 mm parallel-plate geometry at a gap size of 400 μm. Flow sweep measurements were performed over a shear rate range of 0.1 to 1000 s^−1^. Prior to measurement, NH and HA were mixed at the designated ratios and loaded onto the rheometer after mixing. Frequency sweep measurements were conducted over a frequency range of 0.1-10 Hz at a constant strain of 1%.

### 4.5 *In vitro* release profile

STS-loaded NH was prepared by swelling lyophilized microgels in a 790.6 mM STS solution overnight. Release studies were performed using a 24-well transwell system at 37°C. STS-loaded NH or free STS solution (50 μL each) were placed in transwell inserts, with 500 μL of PBS (1×, pH 7.4) added to each well as the release medium. At predetermined time points, the entire release medium was collected and replaced with fresh PBS. The concentration of released STS was quantified by measuring absorbance at 216 nm using a UV-Vis spectrophotometer. All measurements were performed in triplicate (n = 3).

### 4.6 Intracellular ROS assay

Intracellular ROS levels were measured using the DCFDA/H2DCFDA Cellular ROS Assay Kit (ab113851; Abcam, Cambridge, UK) according to the manufacturer's protocol, with analysis performed by fluorescence microscopy and microplate reader. HEI-OC1 cells were seeded into 6-well plates and allowed to attach overnight. Cells were assigned to four groups: control (untreated), cisplatin (20 μM), cisplatin + STS solution, and cisplatin + STS-loaded NH@HA. For STS-treated groups, STS (10 mM) was administered for 2 h prior to cisplatin exposure. STS-loaded NH@HA formulations were applied using transwell inserts (0.8 μm pore size). Cells were maintained under standard culture conditions (33°C, 10% CO_2_) for a total treatment duration of 24 h. Following treatment, cells were washed with phosphate-buffered saline (PBS) and incubated with 25 μM DCFDA in serum-free medium for 45 min at 33°C in the dark. After incubation, cells were washed and subjected to ROS measurements. For qualitative visualization of intracellular ROS, DCF fluorescence was imaged using a fluorescence microscope equipped with a FITC filter set (excitation/emission: ∼485/535 nm). For quantitative analysis, DCF fluorescence was measured using a microplate reader at an excitation wavelength of 485 nm and an emission wavelength of 535 nm.

### 4.7 Analysis of NOX3 via qPCR and western blot

Total RNA was isolated from HEI-OC1 cells using the easy-BLUE™ Total RNA Extraction Kit (iNtRON Biotechnology, Seongnam, Republic of Korea) according to the manufacturer's instructions. Complementary DNA (cDNA) was synthesized from total RNA using the ReverTra Ace™ qPCR RT Master Mix (TOYOBO Co., Ltd., Osaka, Japan) according to the manufacturer's protocol. Quantitative real-time PCR was performed using Power SYBR Green Master Mix (Applied Biosystems, Foster City, CA, USA) with gene-specific primers for NOX3. Amplification was carried out on an ABI 7500 FAST Real-Time PCR System (Applied Biosystems). The relative mRNA expression levels were calculated using the comparative Ct (2^−ΔΔCt^) method and normalized to GAPDH as an internal control. Cells were lysed using RIPA Buffer (LPS Solution, Daejeon, Republic of Korea) supplemented with protease and phosphatase inhibitors. Protein concentrations were determined using the Pierce™ BCA Protein Assay Kit (Thermo Fisher Scientific, Waltham, MA, USA) according to the manufacturer's instructions. Equal amounts of protein were separated by sodium dodecyl sulfate–polyacrylamide gel electrophoresis (SDS-PAGE) and transferred onto nitrocellulose membranes. The membranes were blocked and incubated overnight at 4°C with primary antibodies against NOX3 (1:1000, Proteintech, Rosemont, IL, USA) and β-actin. After incubation with HRP-conjugated secondary antibodies, protein bands were visualized using an enhanced chemiluminescence detection system and analyzed using the ChemiDoc XRS imaging system (Bio-Rad). Band intensities were quantified using Image Lab software and normalized to β-actin.

### 4.8 Animals

All animal experiments were conducted in accordance with the National Research Council Guide for the Care and Use of Laboratory Animals and were approved by the Institutional Animal Care and Use Committee of the Clinical Research Institute, The Catholic University of Korea Daejeon St. Mary's Hospital (approval number: CMCDJ-AP-2025-003). Eight-week-old male BALB/c mice were purchased from Koatech (Pyeongtaek, Korea) and used for all in vivo experiments. Mice were randomly assigned to one of the following four pretreatment groups: (1) Control, IT administration of sterile saline; (2) STS IP, STS (3.5 g/kg, intraperitoneal (IP)); (3) STS IT, IT administration of STS solution (500 mM)(4) STS/NH@HA IT, IT administration of 500 mM STS-loaded NH@HA. In all groups, cisplatin (16 mg/kg, IP) was administered 2h after pretreatment. For IT administration, mice were anesthetized by IP injection of a mixture of Zoletil® 50 (tiletamine–zolazepam, 30 mg/kg; Virbac, Carros, France) and Rompun® (xylazine hydrochloride, 10 mg/kg; Bayer Korea, Seoul, Republic of Korea). Under anesthesia, animals were placed in the supine position with the head oriented upward. A midline cervical incision was made to expose the ventral neck region, and the left auditory bulla was identified under a surgical microscope. A small fenestration was created in the bulla using a fine needle, and a microsyringe was used to slowly deliver sterile saline, STS solution, or STS-loaded NH@HA into the middle ear cavity. Following a volume of ∼10 μL injection into the mouse middle ear cavity, the incision site was sutured.

### 4.9 ABR measurements

Auditory function was assessed by ABR testing at 1, 2, and 3 weeks after IT or systemic administration. ABRs were recorded using a Tucker-Davis Technologies (TDT) System III platform (Tucker Davis Technologies, USA) with tone-burst and click stimulus generation. Under anesthesia, three needle electrodes were inserted subcutaneously at the vertex (active), ventrolateral to the IT-treated left ear (reference), and ventrolateral to the contralateral right ear (ground). Tone-burst stimuli at 8, 16, and 32 kHz, as well as click stimuli, were delivered through a closed-field speaker system coupled to the external auditory canal. ABR thresholds were determined by decreasing the stimulus intensity from 90 to 20 dB SPL. The threshold was defined as the lowest sound intensity that elicited a reproducible ABR waveform.

### 4.10 Quantification of platinum levels in cochlea and liver tissues

Following completion of auditory function testing at 3 weeks after cisplatin administration, cochlear and liver tissues were harvested and weighed. All sample preparation and platinum analysis were performed by the Chungnam National University Shared Experimental Facilities (Daejeon, Korea). Platinum concentrations were determined using ICP-MS and normalized to tissue wet weight. Results are presented as ng/g tissue.

### 4.11 Immunofluorescence staining of cochlear hair cells

Mice were euthanized, and cochleae were carefully dissected and fixed in 4% paraformaldehyde overnight at 4°C on a rotator. Following fixation, cochleae were decalcified in 0.5 M EDTA (pH 8.0; LPS Solution, Korea) at 4°C, with the EDTA solution replaced weekly until complete decalcification was achieved. For whole-mount preparation, the bony capsule, membranous labyrinth, and tectorial membrane were carefully removed under a stereomicroscope using a fine micro-scalpel to expose the organ of Corti. The cochlea was subsequently dissected into apical, middle, and basal turns and maintained in PBS. Samples were permeabilized with 0.5% Triton X-100 in PBS for 15 min at room temperature and incubated with Alexa Fluor 488-conjugated phalloidin (Invitrogen, Carlsbad, CA, USA; 1:1000 dilution) and an anti-myosin VIIa antibody (Bioss, Woburn, MA, USA; Cat. No. bs-7761R; 1:200 dilution) for visualization of cochlear hair cells. After washing with PBS, specimens were mounted on glass slides using VECTASHIELD® mounting medium containing DAPI (Vector Laboratories, Burlingame, CA, USA). Whole-mount specimens were imaged using a confocal laser scanning microscope (LSM 700, Carl Zeiss, Oberkochen, Germany).

## CRediT authorship contribution statement

**Sumin Kim:** Writing – original draft, Methodology, Investigation. **Jee Hyun Kang:** Writing – original draft, Methodology, Investigation. **Seong Su Won:** Methodology, Investigation. **Keum-Jin Yang:** Methodology, Investigation. **Subin Kim:** Methodology. **Dong-Kee Kim:** Writing – review & editing, Supervision, Conceptualization. **Mikyung Shin:** Writing – review & editing, Supervision, Resources, Conceptualization.

## Declaration of competing interests

The authors declare no competing financial interests.

## Data Availability

Data will be made available on request.
