# Supplementary material for: A viscosity-tunable microgel-in-sol phase for localized inner ear delivery of small hydrophilic drug
Source: Mater Today Bio. 2026 Aug 24;40:103590. doi: 10.1016/j.mtbio.2026.103590 (PMC13544082; doi:10.1016/j.mtbio.2026.103590)
Supplement: Supplementary file 1 [file mmc1.docx]

**Supplementary Information**

**A Viscosity-Tunable Microgel-in-sol Phase for Localized Inner Ear Delivery of Small Hydrophilic Drug**

Sumin Kim^†^, Jee Hyun Kang^†^, Seong Su Won, Keum-Jin Yang, Subin Kim, Dong-Kee Kim*, Mikyung Shin^*^

†These authors contributed equally to this work.

*Correspondence to be addressed to: cider12@catholic.ac.kr and mikyungshin@g.skku.edu

**Figure S1.** ^1^H NMR spectrum of NorHA showing characteristic norbornene vinyl proton peaks at 6.2, 6.1, and 5.8 ppm and methylene/bridge protons at 1.1-1.4 ppm (blue shaded region), confirming successful norbornene modification of HA. The HA backbone peaks at 2.8-3.9 ppm are indicated by the gray shaded region.

**Figure S2.** Cell viability assessment of NH using fibroblasts (L929 cell line). (a) Representative live/dead staining images. (b) Quantitative analysis of cell viability determined from images.

**Figure S3.** Representative histological images of the apical, middle, and basal turns of the cochlea following NH injection. Scale bars, 100 μm.

**Figure S4.** Rheological characterization of NH@HA at different [NH] : [HA] volumetric ratios. Storage modulus (grey bars, left y-axis) and tan (δ) (red bars, right y-axis) measured at a fixed frequency of 1 Hz.

**Figure S5.** STS loading process and rheological characterization of NH. (a) Schematic illustration of STS loading into lyophilized NH through reswelling in STS solution. (b) Storage modulus (G′) and loss tangent (tan δ) of NH before lyophilization and after reswelling with STS solution (STS-loaded NH). Comparable G′ and tan δ values indicate that the STS loading process did not substantially alter the mechanical properties of the microgels.

**Figure S6.** Protective effect of STS against cisplatin(CisPt)-induced cytotoxicity at different STS concentrations.

**Figure S7.** In vitro protective effects of HA for HEI-OC1 cells under cisplatin exposure. (a) Cell viability of the cells treated with cisplatin (20 μM) in the absence or presence of HA. (b) Representative DCFH-DA fluorescence images showing intracellular ROS generation (left) and its quantitative analysis (right).

**Figure S8.** A representative western blot image showing NOX3 and β-actin expression in each group.

**Figure S9.** UV-Vis absorption spectra of STS and HA at different concentrations. The red dashed line indicates the absorption peak at the wavelength, 216 nm, used for STS quantification in the in vitro release experiment.

**Figure S10.** Cochlear STS concentrations following IT administration in vivo. Thiosulfate concentrations in cochlear tissues quantified by HPLC-ICP-MS/MS at 1, 3, and 6 h after administration of STS alone (green bars) or STS-loaded NH@HA (red bars).

**Figure S11.** Auditory safety of intratympanic STS at different concentrations. ABR threshold assessment following STS IT administration at a concentration of (a) 100 mM, (b) 500 mM, and (c) 1000 mM. ABR thresholds measured at Week 1 (yellow lines) and Week 3 (orange lines) post-treatment.

**Figure S12.** In vivo evaluation for the effects of NH@HA drug carrier alone under cisplatin exposure. ABR thresholds at 2 and 3 weeks after cisplatin administration of saline (Ctrl) and NH@HA.

**Figure S13.** Quantitative analysis of outer hair cell (OHC) numbers per 100 μm in the apical, middle, and basal turns. Data are presented as mean ± standard deviation (s.d.). n.s. stands for ‘not significant’.

**Supplementary methods**

**Quantification of Cochlear Thiosulfate by HPLC-ICP-MS/MS**

Cochlear concentrations of sodium thiosulfate (STS) were quantified using high-performance liquid chromatography coupled with tandem inductively coupled plasma mass spectrometry (HPLC-ICP-MS/MS), adapted from a previously reported derivatization-based sulfur detection method [1]. Mice were randomly assigned to receive intratympanic injections of either STS solution or STS-loaded NH@HA. Cochlear tissues were harvested at 1, 3, and 6 h after administration. Excised cochleae were immediately weighed and stored at 4 °C until analysis. Sample preparation, derivatization, chromatographic separation, and HPLC–ICP-MS/MS analysis were performed by the GIST Advanced Institute of Analytical Science and Technology (GAIA, Gwangju Institute of Science and Technology, Gwangju, Republic of Korea).

**Quantification of Outer Hair Cells**

Outer hair cell (OHC) quantification was performed using Myosin7a-immunostained cochlear whole-mount images. Myosin7a-positive OHCs were manually counted over a standardized length of 100 μm in the apical, middle, and basal turns using ImageJ software (National Institutes of Health, Bethesda, MD, USA). The average number of OHCs per 100 μm was used for quantitative analysis.

**References**

[1] B. Lafin, R. Somma, B. O-. Pietsch, et al. Trace determination of the hydrogen sulfide biomarker thiosulfate in human urine by HPLC coupled with element selective ICPMS/MS detection. Anal. Chim. Acta 1237 (2023) 340583.
